# Treatment outcomes in primary nummular headache: a systematic review

**DOI:** 10.3389/fphar.2026.1870131

**Published:** 2026-08-20

**Authors:** Aqeel M. Almutairi

**Affiliations:** Department of Neuroimmunology & Headache King Faisal Specialist Hospital & Research Centre, Riyadh, Saudi Arabia

**Keywords:** gabapentin, nummular headache, onabotulinumtoxinA, primary headache disorders, systematic review, treatment outcomes

## Abstract

**Background:**

Nummular headache is a rare primary headache disorder characterized by pain strictly confined to a small, coin-shaped area of the scalp. In the absence of standardized treatment guidelines, management relies on heterogeneous, low-level evidence.

**Objectives:**

To systematically synthesize the available evidence on treatment outcomes in primary nummular headache, focusing on complete remission, clinically meaningful response (≥50% improvement), and treatment tolerability.

**Methods:**

This systematic review followed PRISMA guidelines and was registered in PROSPERO (CRD420261375695). Five electronic databases were searched from inception to March 2026 for studies including ≥5 patients with primary nummular headache according to ICHD criteria. The primary outcome was complete remission; secondary outcomes included ≥50% clinical response and discontinuation due to adverse events. A descriptive synthesis was performed because of substantial clinical and methodological heterogeneity. Risk of bias was assessed with the Newcastle–Ottawa Scale and Joanna Briggs Institute checklists.

**Results:**

Thirty-seven studies (1,012 patients) were included, predominantly case reports, case series, and uncontrolled observational cohorts. OnabotulinumtoxinA was the most robustly evaluated intervention, achieving complete remission in 47.5%–48.8% and ≥50% response in 62.5%–77.4% of patients, with no reported treatment discontinuations due to adverse events. Gabapentin showed complete remission rates of 35.2%–45.5% and ≥50% response rates of 43.7%–65.7%, but was associated with a 21.1% discontinuation rate because of adverse effects. Evidence for amitriptyline, lamotrigine, pregabalin, and other therapies was limited to smaller samples and showed variable efficacy. Outcome definitions varied considerably across studies, and no randomized comparative trials were identified.

**Conclusion:**

Current evidence suggests that onabotulinumtoxinA offers the most favorable efficacy–tolerability profile, while gabapentin is effective but limited by tolerability concerns. The overall evidence base remains predominantly uncontrolled and heterogeneous. Prospective comparative studies with standardized outcome measures are urgently needed to establish definitive treatment guidelines for this disabling headache disorder.

**Systematic Review Registration:**

Identifier CRD420261375695.

## Graphical Abstract

**Figure:**
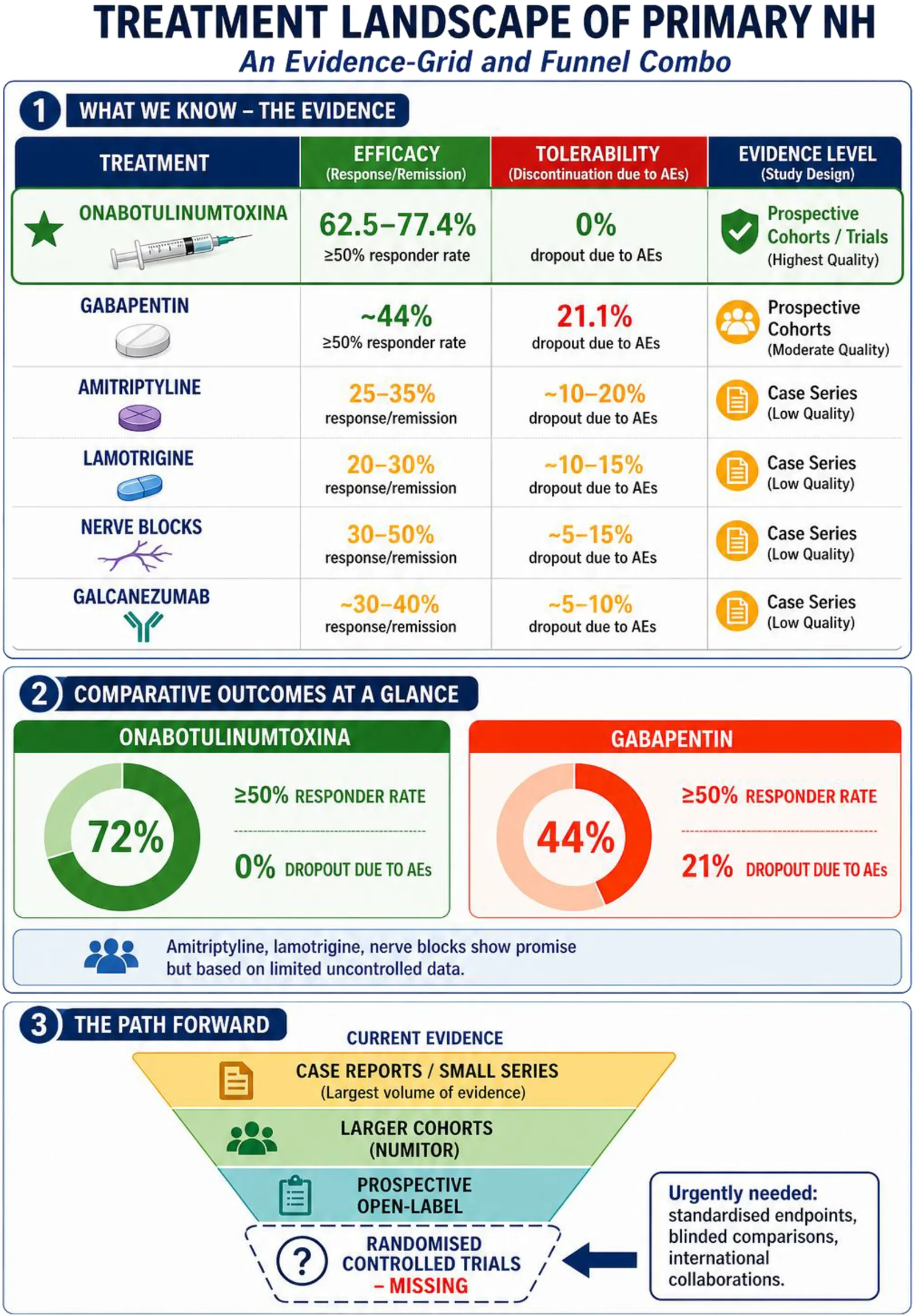
Take‑Home Visual Treatment Landscape of Primary NH: An Evidence-Grid and Funnel Combo: This multi-tier infographic summarizes the current treatment landscape of primary NH by integrating efficacy, tolerability, and evidence quality. The top panel presents an evidence grid comparing major therapies, highlighting onabotulinumtoxinA as having the most favorable efficacy–tolerability profile and highest-quality evidence (prospective cohorts), while gabapentin shows moderate efficacy but reduced tolerability. The middle panel provides a direct visual comparison of treatment outcomes, demonstrating higher response rates and lower dropout with onabotulinumtoxinA (72% response, 0% discontinuation) versus gabapentin (44% response, 21% discontinuation). The bottom panel depicts the hierarchy of available evidence using a funnel model, illustrating the predominance of case reports and small series and the absence of randomized controlled trials (RCTs). The figure highlights a critical evidence gap and underscores the need for standardized endpoints and prospective comparative studies.

## 1 Introduction

Nummular headache (NH) is a relatively recent addition to the spectrum of primary headache disorders, first described by Pareja et al., in 2002 as a distinct clinical entity characterized by pain confined to a small, well-circumscribed, coin-shaped area of the scalp (Pareja et al., 2002). Since its initial description, NH has gained increasing recognition due to its unique topography and clinical behavior, distinguishing it from other primary headaches such as migraine or tension-type headache (Headache Classification Committee of the International Headache Society IHS, 2013). Initially included in the appendix of the second edition of the International Classification of Headache Disorders (ICHD-2) as a research entity, NH was later incorporated into the main classification in ICHD-3 under “other primary headache disorders,” reflecting growing evidence supporting its validity as a discrete condition (Headache Classification Subcommittee of the International Headache Society, 2004; Cuadrado et al., 2018). Despite this progress, NH remains underdiagnosed, and its pathophysiology is not fully understood, although current evidence suggests a predominant role of peripheral epicranial structures (Trigo et al., 2019).

The management of NH remains challenging due to the absence of standardized treatment guidelines and the heterogeneity of available evidence. A wide range of pharmacological and interventional therapies has been reported, targeting both central and peripheral mechanisms. Antiepileptic drugs such as gabapentin, pregabalin, carbamazepine, and lamotrigine are commonly used and are thought to modulate neuronal excitability through calcium channel inhibition or sodium channel blockade, thereby reducing abnormal nociceptive signaling (García-Iglesias et al., 2022; Janjua and Singh, 2020). Tricyclic antidepressants, including amitriptyline, exert analgesic effects via modulation of serotonergic and noradrenergic pathways (García-Iglesias et al., 2023). OnabotulinumtoxinA has emerged as a promising therapy, likely acting through inhibition of peripheral neurotransmitter release and reduction of sensitization of nociceptive fibers (García-Iglesias et al., 2022). Similarly, local anesthetic injections and nerve blocks target peripheral pain generators by interrupting transmission in epicranial nerves (Guerrero et al., 2012). Other therapies, including nonsteroidal anti-inflammatory drugs, beta-blockers, calcitonin gene-related peptide (CGRP) monoclonal antibodies (e.g., galcanezumab), and even surgical interventions such as resection of underlying vascular anomalies, have been reported with variable success (Cuadrado et al., 2018; García-Iglesias et al., 2023; Baldelli et al., 2020). The diversity of these treatments reflects the uncertainty regarding the underlying mechanisms of NH and suggests that both peripheral and central pathways may be involved.

Several narrative reviews and case series have attempted to summarize the available evidence on NH treatment; however, the literature remains largely composed of case reports, small case series, and retrospective cohorts (García-Iglesias et al., 2021). While some studies suggest favorable outcomes with agents such as gabapentin or onabotulinumtoxinA, the reported efficacy varies widely, and high remission rates are frequently observed in low-level evidence, raising concerns about potential overestimation of treatment effects (Cuadrado et al., 2018). To date, there is a lack of structured descriptive synthesis evaluating treatment outcomes across different therapeutic modalities. Most previous reviews have not systematically assessed clinically meaningful endpoints such as complete remission or ≥50% reduction in symptoms, nor have they adequately addressed treatment tolerability and safety profiles.

Given the heterogeneity of available treatments and the predominance of low-quality evidence, there is a clear need for a systematic and quantitative evaluation of therapeutic outcomes in NH. This systematic review and descriptive synthesis aims to synthesize the existing literature to provide a comprehensive assessment of treatment effectiveness, focusing on clinically relevant endpoints including complete remission and meaningful symptom improvement. By integrating data across studies, this work seeks to distinguish between apparent efficacy driven by small case reports and more reliable outcomes derived from larger cohorts. Additionally, this study aims to compare different therapeutic classes and explore their relative effectiveness and safety, thereby providing clinicians with evidence-based insights to guide management decisions. Ultimately, this study addresses a critical gap in the literature and contributes to a more structured understanding of treatment strategies in primary nummular headache, with potential implications for both clinical practice and future research.

## 2 Methods

This study was conducted following the Cochrane Handbook for Systematic Reviews of Interventions (Cochrane, 2024). The results were reported as specified by the Preferred Reporting Items for Systematic Reviews and Meta-Analyses (PRISMA) statement (Pag et al., 2021). Additionally, this study has been registered with PROSPERO under the identification number (CRD420261375695).

### 2.1 Information sources and search strategy

The literature search used five electronic databases: Cochrane, PubMed, Embase, Web of Science, and Scopus. The search targeted literature from inception to 30 March 2026. The strategy employed utilized keywords such as (Supplementary Table S1) along with their pertinent synonyms.

**TABLE 1:** Comparison of diagnostic criteria for nummular headache.

Feature	(Original description) (Pareja et al., 2002)	(Appendix A13.7.1) (Headache Classification Subcommittee of the International Headache Society, 2004)	(Beta) (Headache Classification Committee of the International Headache Society IHS, 2013)	(Final) (ICHD-3, 2018)
Pain location	Small, circumscribed round/elliptical area of the head	Small circumscribed area of the head	Pain is exclusively localized to a small area of the scalp	Same as ICHD-3β
Size	Typically 1–6 cm	Typically 2–6 cm	1–6 cm	1–6 cm
Shape	Round or elliptical	Round or elliptical	Sharply contoured, fixed in size and shape	Same as ICHD-3β
Temporal pattern	Chronic or remitting; continuous or intermittent	Chronic, with or without remissions	No fixed duration requirement; may be continuous or intermittent	Same as ICHD-3β
Exacerbations	Possible (lancinating exacerbations described)	Not included in the criteria	Not required but acknowledged	Not required but acknowledged
Sensory changes	Frequently described (hypoesthesia, dysesthesia, allodynia)	Not included	Common but not required	Common but not required
Trophic changes	Occasionally reported	Not mentioned	Not included	Not included
Classification	Proposed as a new entity	Included in the appendix (research criteria)	Included in main classification (Part 1: primary headaches)	Maintained in the main classification
Secondary forms	Not emphasized	Not emphasized	Recognized in literature	Recognized; secondary causes must be excluded
Exclusion criteria	No underlying structural lesion	Not attributed to another disorder	Not better accounted for by another diagnosis	Diagnosis requires exclusion of secondary causes

### 2.2 Eligibility criteria

#### 2.2.1 Inclusion & exclusion criteria (PICO)

1. Population: Patients diagnosed with primary NH according to ICHD-2, ICHD-3β, ICHD-3, or Pareja’s 2002 criteria (Table 1). Exclude: Studies focusing solely on secondary NH, post-traumatic NH, or pediatric populations. If a study includes a mix, data for primary NH must be extractable separately.
2. Intervention/Exposure: Any pharmacological (e.g., gabapentin, onabotulinumtoxinA, NSAIDs, amitriptyline, carbamazepine) or interventional (e.g., nerve blocks) treatment administered with the intent of improving NH.
3. Comparator: No comparator is required for a single-arm proportional meta-analysis. If studies with comparator groups exist, they will be analyzed separately.
4. Outcomes:
  - *Primary:* Complete remission was operationally defined as complete pain disappearance, asymptomatic status, or complete resolution/remission of NH pain as reported by the original study authors. When studies used terms such as “optimal response” rather than “complete remission,” the original definition was retained and clearly described.
  - Secondary:
    - Clinically meaningful response was operationally defined as a ≥50% reduction in headache days, headache frequency, or pain intensity. When a standardized ≥50% response endpoint was not available, author-defined “good,” “adequate,” or “optimal” response was extracted and reported according to the terminology used in the original study.
    - Mean change in headache days per month.
    - Mean change in pain intensity (e.g., on VAS/NRS).
    - Proportion of patients discontinuing treatment due to adverse events.
5. Study Design: Prospective or retrospective observational studies (cohort studies, case series with ≥5 patients) that report original data on treatment outcomes for primary NH. Case reports (<5 patients) will be excluded from the primary meta-analysis but can be listed in a supplementary table in the systematic review.

#### 2.2.2 Screening and data management

The screening process was carried out in two distinct phases. The first phase involved the initial screening of titles and abstracts to identify potentially relevant studies. In the second phase, full-text articles of selected studies were thoroughly reviewed to confirm eligibility. Rayyan software was used to facilitate the screening process. One reviewer independently conducted the eligibility assessment (Ouzzani et al., 2016).

### 2.3 Data extraction

Data extraction was independently performed using standardized data extraction sheets to ensure consistency and accuracy. The extracted data included study characteristics such as study identification (Study ID), country, study design, clinical setting, and diagnostic criteria used for nummular headache. Additionally, sample-related variables were collected, including total nummular headache cohort size and the number of patients with primary nummular headache included in the treatment analysis. Patient baseline characteristics were also recorded, including age at treatment initiation (mean ± SD), sex distribution (percentage of females), and pain duration at baseline (mean ± SD). Treatment-related variables included the type of treatment evaluated, dosage regimen, and duration of follow-up (mean and/or range). Furthermore, an overlap check was conducted based on study center and recruitment period to identify and avoid potential duplication of patient populations. Because several included studies originated from Spanish headache centers with partially overlapping recruitment periods, a predefined overlap assessment was performed to reduce the risk of duplicate patient inclusion. Studies were compared according to study center, author group, recruitment period, sample size, cohort description, and reported treatment outcomes. Particular attention was given to cohorts from Valladolid, Alcorcón, Madrid, and other Spanish headache units. When two or more studies originated from the same center or author group and had overlapping recruitment periods, the possibility of patient overlap was considered. In such cases, the largest or most comprehensive cohort was prioritized for descriptive interpretation, whereas smaller or potentially overlapping reports were retained only when they provided unique treatment-specific, follow-up, or phenotypic information.

### 2.4 Risk of bias assessment

Risk of bias assessment was independently conducted by using appropriate standardized tools according to the study design. For cohort and case-control studies, the Newcastle–Ottawa Scale (NOS) was applied to evaluate methodological quality across three domains: selection of study groups, comparability of groups, and ascertainment of exposure or outcomes. Each study was assigned a star-based score, and overall quality was categorized as low, moderate, or high risk of bias based on the total score. For case reports, the Joanna Briggs Institute (JBI) Critical Appraisal Checklist for Case Reports was utilized. This tool assesses key methodological components, including a clear description of patient demographics, clinical history, and timeline, diagnostic methods, intervention details, post-intervention clinical condition, adverse events, and takeaway lessons. Each item was evaluated as “yes,” “no,” “unclear,” or “not applicable,” and an overall judgment regarding methodological quality was made accordingly (Guald et al., 2026; Hilto, 2024).

### 2.5 Data synthesis and handling of quantitative data

A quantitative synthesis was initially considered during protocol development. However, formal meta-analysis was not performed because of substantial clinical and methodological heterogeneity across the included studies. Outcome definitions varied considerably across reports, particularly for complete remission and clinically meaningful response. In addition, most studies were uncontrolled, comparator groups were absent, sample sizes were small, variance estimates such as standard deviations or confidence intervals were frequently unavailable, and potential overlap between some cohorts could not be fully excluded.

Therefore, treatment outcomes were synthesized descriptively. Dichotomous outcomes, including complete remission, clinically meaningful response, and discontinuation due to adverse events, were summarized as numerators, denominators, and percentages when extractable. Continuous outcomes, including changes in headache days per month and pain intensity scores, were reported as presented by the original study authors. No pooled effect sizes, summary risk estimates, forest plots, or network meta-analysis were calculated.

Case reports and very small case series were included only for narrative synthesis and were not interpreted as providing generalizable estimates of treatment efficacy. When single-case reports showed complete remission or 100% response, these findings were considered hypothesis-generating only.

## 3 Results

### 3.1 Search and screening

**FIGURE 1:**
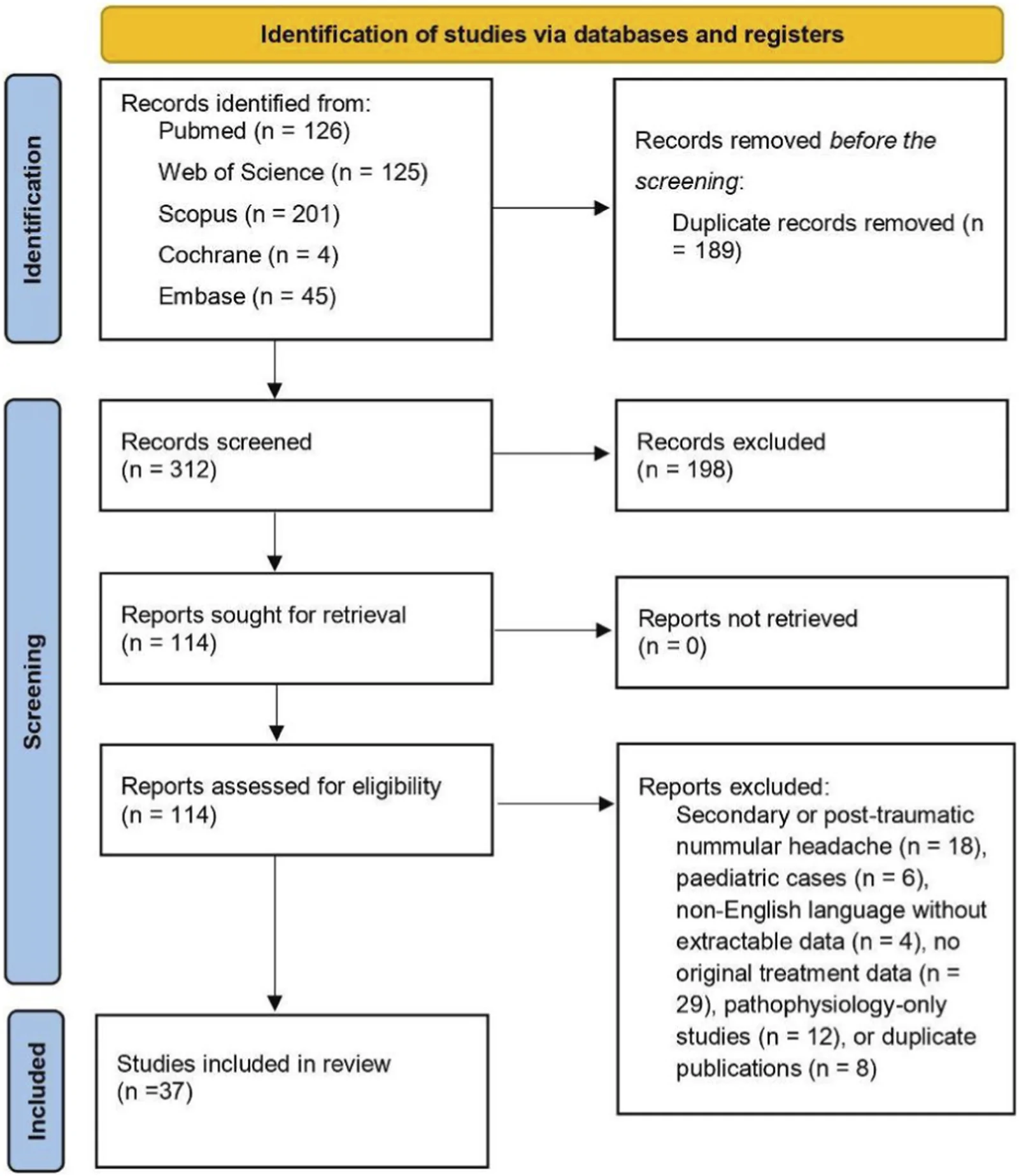
Prisma flow diagram.

A systematic literature search was conducted across five electronic databases: PubMed (n = 126), Scopus (n = 201), Web of Science (n = 125), Cochrane Library (n = 4), and Embase (n = 45), covering the period from January 2002 to March 2026. After removing 189 duplicate records, 312 unique records remained. These were screened by title and abstract, resulting in the exclusion of 198 records that did not meet the eligibility criteria (e.g., non-primary headache, paediatric populations, non-treatment studies). The remaining 114 full-text articles were assessed for eligibility against the predefined PICOS criteria. Of these, 77 were excluded for reasons including secondary or post-traumatic nummular headache (n = 18), paediatric cases (n = 6), non-English language without extractable data (n = 4), no original treatment data (n = 29), pathophysiology-only studies (n = 12), or duplicate publications (n = 8). Consequently, 37 studies were included in the systematic review (with a total of 1,012 patients) (Trigo et al., 2019; García-Iglesias et al., 2022; García-Iglesias et al., 2023; Guerrero et al., 2012; Yamazaki and Kobatake, 2011; Thomas et al., 2020; Tereshko et al., 2023; Ruscheweyh et al., 2010; Rodríguez et al., 2015; Rocha-Filho, 2011; Rammohan et al., 2016; Porta-Etessam et al., 2010; Pellesi et al., 2020; Pareja et al., 2004; Mulero et al., 2013; Mathew et al., 2008; Man et al., 2012; López-Ruiz et al., 2014; López-Mesonero et al., 2014; López-Bravo et al., 2022; Liu and Wei, 2018; Jiang et al., 2019; Iwanowski et al., 2014; Irimia et al., 2013; Hall and Vajramani, 2021; Guerrero et al., 2011; García-Azorín et al., 2019; Danno et al., 2013; Clar-de-Alba et al., 2020; Camacho-Velasquez, 2016; Robbins and Grosberg, 2009; Barón et al., 2015; Patel et al., 2020; Evans and Pareja, 2005; Chen et al., 2013; Chirchiglia et al., 2020; Panda et al., 2021) (Figure 1).

### 3.2 Study characteristics

The 37 included studies comprised a range of designs, reflecting the evolving understanding of nummular headache since its initial description. Geographically, most studies originated from Spain (n = 14), followed by China (n = 4), Italy (n = 3), India (n = 3), and the remaining from the USA, Japan, Germany, the UK, Brazil, Poland, and Taiwan. Diagnostic criteria varied according to publication date: 12 studies used ICHD-2 (2004), 7 used ICHD-3β (2013), 9 used ICHD-3 (2018), and one (Pareja, 2004) applied the original descriptive criteria. Study designs included 19 case reports, 12 case series (2–72 patients), and 6 observational cohort studies, with the largest cohorts comprising 225 (Trigo, 2019) and 183 (García-Iglesias et al., 2023) patients. Across all studies, 1,012 patients were included in the primary analysis for treatment, with a median age at treatment of 49 years (range 11–86) and a female predominance (median 64.4%). Baseline pain duration was highly variable, from 1 month to over 50 years, with a median of approximately 10 months in the larger series.

Treatment evaluation was heterogeneous, reflecting the absence of standardised guidelines. The most frequently assessed preventive interventions were gabapentin (evaluated in 12 studies, dosages 100–1,800 mg/day), onabotulinumtoxinA (8 studies, typically 25 U divided over 5–10 injection sites), amitriptyline (7 studies, 10–50 mg/day), and carbamazepine/oxcarbazepine (6 studies, 200–900 mg/day). Other medications included topiramate, pregabalin, lamotrigine, and Neurotropin. Follow-up durations ranged from 2 weeks to 20 years; the longest follow-up was reported in the Spanish long-term series (Clar-de-Alba, 2020; median 12 years). Most studies were single-centre; only two were multicentre (Guerrero, 2011; Pellesi, 2020). Quality assessment using the Joanna Briggs Institute checklists for case reports and series yielded median scores of 7.5/8 and 7.5/10, respectively, indicating generally complete reporting. For the six cohort studies evaluated with the Newcastle–Ottawa Scale, scores ranged from 5 to 6 out of 9 stars, reflecting adequate selection and outcome ascertainment but a lack of non-exposed comparison groups. Overall, the included studies provide a rich but methodologically heterogeneous evidence base, with larger contemporary cohorts employing prospective registries and standardised outcome definitions, while earlier studies contributed essential descriptive data on this rare headache disorder (Table 2).

**TABLE 2:** Study characteristics.

Study ID	Country	Study design	Setting	Diagnostic criteria	Total NH cohort (n)	Primary NH for Tx analysis (n)	Age at Tx (mean, SD)	Sex (F, %)	Pain duration at baseline (mean, SD)	Treatment(s) evaluated	Dosage regimen	Follow-up duration (mean, range)	Overlap check (center/period)	Tool of QA	Score of QA
Barón et al. (2015)	Spain	Case report	Headache unit	ICHD-III beta	1	1	57 (case age)	100%	NR	Almotriptan (symptomatic), Topiramate (preventive)	Almotriptan (dose NS), Topiramate 100 mg/day	NR	None	JBI for Case Reports	7/8
Camacho-Velasquez et al. (2016)	Spain	Case report	Neurology clinic	ICHD-II	1	1	47 (case age)	0%	3 months	Pregabalin, Topical corticosteroid	Pregabalin 75 mg/day	NR	None	JBI for Case Reports	8/8
Chen et al. (2013)	Taiwan	Case series	Neurology department, tertiary hospital	ICHD-II	30	5	48.5 ± 3.2	100%	1–10 years (range)	Gabapentin, NSAIDs	Gabapentin 100–300 mg/day; NSAIDs (type NS)	1 year (for some cases)	Single center (Kaohsiung Chang Gung Memorial Hospital), period: 2007–2012	JBI for Case Series	7/10
Chirchiglia et al. (2020)	Italy	Case report	University hospital	ICHD-III beta	1	1	57 (case age)	100%	Long history, NS	Topiramate, Palmitoylethanolamide (PEA)	Topiramate 50–100 mg/day; PEA 600 mg/day	3 years	None	JBI for Case Reports	7/8
Clar-de-Alba et al. (2020)	Spain	Retrospective cohort	Headache unit, tertiary hospital	ICHD-II	83	83	46 (mean at onset)	51%	Median 7 months (0–144)	Gabapentin, Tricyclics, Topiramate, Carbamazepine, Opioids, OnabotulinumtoxinA, Anesthetic blocks	Variable doses; OnabotA: 10 U x 10 points	Median 12 years (Baldelli et al., 2020; García-Iglesias et al., 2021; Cochrane, 2024; Pag et al., 2021; Ouzzani et al., 2016; Guald et al., 2026)	Single center (Hospital Universitario Fundación Alcorcón), period: 2003–2018	NOS (cohort)	5/9
Danno et al. (2013)	Japan	Case series	NR	ICHD-II	3	3	55.7 (range 39–71)	33.30%	1 week to >1 year	Neurotropin (NTP)	16 units/day	1–3 months	None	JBI for Case Reports (adapted)	6/8
Evans et al. (2005)	USA	Case report	Neurology clinic	ICHD-II (implied)	1	1	45 (case age)	100%	3 months	Gabapentin	100 mg PRN	NR	None	JBI for Case Reports	7/8
Garcia-Azorín et al. (2019)	Spain	Prospective cohort	Headache unit, tertiary hospital	ICHD-3 beta	53	53	Median 54 (IQR 41–72)	67.90%	Median 11 months (IQR 6–33)	OnabotulinumtoxinA	25 U divided over 5 points	24 weeks	Single center (Hospital Clinico Universitario de Valladolid), period: 2017–2019	NOS (cohort)	6/9
García-Iglesias et al., (2023)	Spain	Ambispective cohort (NUMITOR)	Headache unit, tertiary hospital	ICHD-3	282 (screened), 183 (final)	183	49.5 ± 16.8	60.70%	Median 7.5 months (IQR 3–14.2)	Gabapentin, OnabotA, Amitriptyline, Lamotrigine, Pregabalin, others	Variable; OnabotA: 25 U (5U x 5 points)	12 weeks (for response)	Single center (Hospital Clinico Universitario de Valladolid), period: 2002–2022 (retrospective analysis of prospectively collected data)	NOS (cohort)	6/9
Garcia-Iglesias et al. (2023)	Spain	Observational, ambispective cohort	Headache unit, tertiary hospital	ICHD-3	168	168	49 (median at diagnosis, IQR 35–64)	63.70%	Median 10 months (IQR 5–24)	Gabapentin, OnabotA, Amitriptyline, Lamotrigine, NSAIDs, Paracetamol	Variable; OnabotA: 25 U (5U x 5 points)	Median 80.5 months (55–118.5)	Single center (Hospital Clinico Universitario de Valladolid), period: 2008–2021	NOS (cohort)	6/9
Guerrero et al. (2011)	Spain	Case series	Headache clinics, 2 tertiary hospitals	ICHD-II	70	6	40.8 ± 19.1	66.70%	2 months to 50 years (range)	Gabapentin, Carbamazepine	Gabapentin 800–1,200 mg/day; Carbamazepine 400 mg/day	NR	Two centers (Valladolid, Madrid), period: 2008–2010	JBI for Case Series	7/10
Guerrero et al. (2012)	Spain	Case series	Headache unit, tertiary hospital	ICHD-II	72 (80 areas)	72	47.3 ± 18.5	61.10%	NR	Gabapentin, Lamotrigine	Gabapentin 800–1800 mg/day; Lamotrigine 100–200 mg/day	NR	Single center (Hospital Clinico Universitario de Valladolid), period: 2008–2011	JBI for Case Series	9/10
Hall et al. (2021)	United Kingdom	Case report	Neurosurgery center	ICHD (unspecified edition)	1	1	45 (case age)	0%	4 years	Percutaneous Electrical Nerve Stimulation (PENS)	20 or 50 mm electrode, 25 min stimulation	4 years (cumulative)	None	JBI for Case Reports	8/8
Irimia et al. (2013)	Spain	Case report	Neurology clinic	ICHD-II	1	1	33 (case age)	100%	1 year	OnabotulinumtoxinA	100 U (for headache and alopecia)	3 months (follow-up reported)	None	JBI for Case Reports	8/8
Iwanowski et al. (2014)	Poland	Case report	Department of neurology	ICHD-II	1	1	61 (case age)	100%	1 year	Gabapentin, Mianserin, Bupivacaine blocks	Gabapentin (dose NS); Mianserin (dose NS); Bupivacaine injection	Up to 3 months	None	JBI for Case Reports	6/8
Jiang et al. (2019)	China	Case report + narrative review	Neurology department	ICHD-3	2 (cases) + 312 (lit. review)	2	48 and 72 (case ages)	50%	11 and 10+ years	Metoprolol	25–37.5 mg/day	6–12 months	None	JBI for Case Reports (adapted)	7/8
Liu et al. (2018)	China	Case series	Headache clinic	ICHD (unspecified)	3	3	52.7 (range 38–74)	66.70%	2 months to 20 years	Gabapentin, Acupuncture, Ibuprofen	Gabapentin (dose NS)	Up to 2 weeks	None	JBI for Case Reports (adapted)	6/8
López-Bravo et al. (2022)	Spain	Case report	Neurology Department	ICHD-3	1	1	64 (case age)	0%	8 years	Galcanezumab	240 mg loading, then 120 mg/month	12 months	None	JBI for Case Reports	8/8
Lopez-Mesonero et al. (2014)	Spain	Case report	Neurology department	ICHD-II/III (implied)	1	1	41 (case age)	100%	1 year	None (refused)	-	-	None	JBI for Case Reports	7/8
López-Ruiz et al. (2014)	Spain	Case series	Outpatient neurology clinic	ICHD-III beta	2	2	63.5 (range 60–67)	0%	4–25 years	Triptans (zolmitriptan, sumatriptan), Surgical resection (Case 1)	Zolmitriptan, sumatriptan (doses NS)	8 months (Case 1)	None	JBI for Case Reports (adapted)	8/8
Man et al. (2012)	China	Case report	Department of neurology	ICHD-II	1	1	59 (case age)	0%	1 month	Carbamazepine	200–400 mg/day	6 months	None	JBI for Case Reports	7/8
Mathew et al. (2008)	United States of America	Case series	Headache clinic	ICHD-II	4	4	35–58 (range)	100%	Mean 3.75 years	OnabotulinumtoxinA	25 U divided over 10 sites	14 weeks (average per cycle)	None	JBI for Case Series	8/10
Mulero et al. (2013)	Spain	Case series	Neurology department	ICHD (unspecified)	3	3	33.7 (range 21–45)	0%	4 months to 15 years	Gabapentin, Indomethacin	Gabapentin 1,200 mg/day; Indomethacin (dose NS)	NR	None	JBI for Case Reports (adapted)	6/8
Panda et al. (2021)	India	Case report	Pediatrics, tertiary hospital	ICHD-3	1	1	11 (case age)	100%	6 months	Carbamazepine, Gabapentin	Carbamazepine 30 mg/kg/day; Gabapentin 600 mg/day	3 months	None	JBI for Case Reports	7/8
Pareja et al. (2004)	Spain	Case series	Neurological clinic	ICHD (1st ed, adapted)	14	14	38 (mean at onset)	78.60%	1 month to 5 years (mostly)	Paracetamol (when needed)	Standard oral doses	NR	Single center (Fundación Hospital Alcorcón), period: 2002–2003	JBI for Case Series	8/10
Patel et al. (2020)	India	Case report	Tertiary care hospital	ICHD-III beta	1	1	54 (case age)	0%	4 years	Oxcarbazepine	900 mg/day	1 year	None	JBI for Case Reports	7/8
Pellesi et al. (2020)	Italy	Retro-prospective cohort	7 Tertiary headache centres	ICHD-3	19	19	47 (mean at diagnosis)	42.10%	8.2 years (mean)	NSAIDs, Pregabalin, Amitriptyline, Gabapentin, OnabotA, others	Variable; Pregabalin 150–450 mg/day; Amitriptyline 10–50 mg/day; Gabapentin 900 mg/day	NR	Multi-center, period: 2014–2017	NOS (cohort)	5/9
Porta-Etessam et al. (2010)	Spain	Case report	NR	ICHD (unspecified)	1	1	52 (case age)	100%	2 months	None (spontaneous improvement)	-	4 months	None	JBI for Case Reports	7/8
Rammohan et al. (2016)	India	Prospective, observational	Medicine & neurology OPD, tertiary hospital	ICHD-3 beta	29	29	47.6 ± 11.9	65.50%	Mean 24.2 months (3–60)	Not a treatment study (descriptive only)	-	-	Single center, period: 2013–2015	JBI for Case Series	7/10
Robbins et al. (2009)	United States of America	Case report	Headache center	ICHD (unspecified)	1	1	40 (case age)	100%	3 years	Cyclobenzaprine	5 mg TID	NR	None	JBI for Case Reports	7/8
Rocha-Filho et al. (2011)	Brazil	Case report	NR	ICHD-II	1	1	52 (case age)	0%	4 years	Subcutaneous lidocaine	Local application, dose NS	NR	None	JBI for Case Reports	7/8
Rodriguez et al. (2015)	Spain	Case report	Headache unit, tertiary hospital	ICHD-3 beta	1	1	14 (case age)	100%	3 years	Gabapentin	900 mg/day	20 months	None	JBI for Case Reports	8/8
Ruscheweyh et al. (2010)	Germany	Case series	Supraregional headache clinic	ICHD-II	6	6	38–60 (range)	66.70%	2 months to 2 years	Amitriptyline, Duloxetine, Gabapentin, Topiramate, OnabotA	Variable; Gabapentin 300–900 mg/day	NR	None	JBI for Case Series	7/10
Tereshko (2023)	Italy	Case report	Headache outpatient clinic	ICHD-3	1	1	58 (case age)	100%	1 year (since 2019)	Ketogenic Diet (VLCKD), OnabotulinumtoxinA	VLCKD 730 Kcal/day for 6 months; OnabotA 50 U (5U x 10 points)	7+ months (diet), 3–4 months per BoNT/A cycle	None	JBI for Case Reports	8/8
Thomas (2020)	USA	Case report	Dental/Orofacial pain clinic	ICHD-3	1	1	26 (case age)	100%	>10 years	Gabapentin	600–900 mg/day	NR	None	JBI for Case Reports	8/8
Trigo (2019)	Spain	Observational cohort	Headache unit, tertiary hospital	ICHD-II/III	225	225	49.6 ± 18.3	64.40%	Median 10 months (IQR 5–24)	Gabapentin, Lamotrigine, Amitriptyline, Pregabalin, OnabotA	Variable; Gabapentin most common 800 mg/day	NR (cross-sectional)	Single center (Hospital Clinico Universitario de Valladolid), period: 2008–2018	NOS (cohort)	5/9
Yamazaki and Kobatake (2011)	Japan	Case report	NR	ICHD-II	1	1	28 (case age)	0%	2 months	Neurotropin (NTP)	16 units/day	2 months (treatment duration)	None	JBI for Case Reports	7/8

Abbreviations: F, female; ICHD, International Classification of Headache Disorders; IQR, interquartile range; NH, nummular headache; NS, NR; OnabotA, onabotulinumtoxinA; PEA, palmitoylethanolamide; PRN, as needed; SD, standard deviation; TID, three times daily; Tx, treatment; VLCKD, very-low-calorie ketogenic diet.

### 3.3 Risk of bias assessment

A quality assessment of the 37 included studies was performed using appropriate critical appraisal tools. Case reports and case series were evaluated with the Joanna Briggs Institute (JBI) checklists, while observational cohort studies were assessed using the Newcastle–Ottawa Scale (NOS). Among the 28 case reports or small case series, JBI scores ranged from 6/8 to 8/8 for case reports (median 7.5) and from 7/10 to 9/10 for case series (median 7.5). The highest scores were achieved by studies with comprehensive clinical descriptions, clear treatment documentation, and adequate follow-up. For the six observational cohort studies evaluated with the NOS, scores ranged from 5/9 to 6/9 (median 6). These studies consistently demonstrated good selection of exposed cohorts and secure ascertainment of exposure, but lacked non-exposed comparison groups, limiting comparability. Overall, the methodological quality of the included studies was moderate to high, reflecting detailed case documentation and, in the larger cohorts, rigorous prospective data collection. Common limitations across study types included the absence of statistical comparisons in case series, variable follow-up durations, and the inherent risk of selection bias in retrospective analyses (Supplementary Table S2).

### 3.4 Outcomes

#### 3.4.1 Primary outcome: complete remission

Complete remission was reported in 15 studies, with marked variation in definition and frequency across treatments. Among patients treated with gabapentin, the proportion achieving complete remission ranged from 35.2% (25/71) in the NUMITOR cohort (García-Iglesias et al., 2023) to 45.5% (15/33) after 5 years of follow-up (Clar-de-Alba, 2020), with small case series reporting 100% complete remission (Rodriguez, 2015; Chen, 2013; Mulero, 2013). OnabotulinumtoxinA yielded complete remission in 47.5% (19/40) of patients in the NUMITOR study, defined as a >75% reduction in headache days, and achieved complete resolution in individual case reports (Tereshko, 2023). Neurotropin was associated with complete remission in two of three patients (66.7%) in one series (Danno, 2013) and in a single case report (Yamazaki, 2011). Complete remission was also documented for metoprolol (Jiang, 2019), galcanezumab (López-Bravo, 2022), oxcarbazepine (Patel, 2020), carbamazepine (Man, 2012), indomethacin (Mulero, 2013), and lidocaine injection (Rocha-Filho, 2011), each in a single case. Surgical resection of a superficial artery aneurysm resulted in complete and sustained remission in one patient (López-Ruiz, 2014). Outcome definitions were heterogeneous across the included studies. Therefore, complete remission and clinically meaningful response were reported according to the operational definitions specified in the Methods, while preserving the original definitions used by individual studies where relevant (Table 3; Supplementary Table S3; Supplementary Figure S1).

**TABLE 3:** Complete remission by treatment.

Treatment	Treated	Remission	No remission	Rate (%)
Gabapentin	108	44	64	40.7
OnabotulinumtoxinA	41	20	21	48.8
Neurotropin	4	3	1	75
Metoprolol	1	1	0	100
Galcanezumab	1	1	0	100
Oxcarbazepine	1	1	0	100
Carbamazepine	1	1	0	100
Indomethacin	1	1	0	100
Lidocaine injection	1	1	0	100
Surgery (aneurysm resection)	1	1	0	100

#### 3.4.2 Secondary outcome: clinically meaningful response (≥50% improvement)

A ≥50% reduction in headache frequency or intensity was reported in 19 studies, with responder rates varying by treatment. For gabapentin, the ≥50% responder rate was 43.7% (31/71) in the NUMITOR study (Garcia-Azorín et al., 2023) and 67.7% (86/127) among patients receiving any oral preventive in the Trigo, 2019 series. OnabotulinumtoxinA demonstrated the highest responder rates: 62.5% (25/40) in the NUMITOR study and 77.4% (41/53) in the prospective open-label trial by García-Azorín et al., (2019), with all treated patients in smaller case series achieving ≥50% improvement (Tereshko, 2023; Irimia, 2013; Mathew, 2008). Pregabalin (40%, 2/5) and amitriptyline (66.7%, 4/6) showed moderate efficacy in the Italian multicentre series (Pellesi, 2020). Lamotrigine was associated with a 34.8% (8/23) responder rate in the NUMITOR study. Single cases with ≥50% improvement were documented for carbamazepine, topiramate, ketogenic diet, PENS, and metoprolol (Table 4; Supplementary Table S4; Supplementary Figure S2).

**TABLE 4:** ≥50% clinical response by treatment.

Treatment	Treated	Responders	Non-responders	Rate (%)
Gabapentin	178	117	61	65.7
OnabotulinumtoxinA	99	72	27	72.7
Lamotrigine	23	8	15	34.8
Amitriptyline	6	4	2	66.7
Pregabalin	6	3	3	50
Topiramate	2	2	0	100
Carbamazepine	1	1	0	100
Anesthetic blocks	1	1	0	100
Ketogenic diet (VLCKD)	1	1	0	100
PENS	1	1	0	100
Metoprolol	1	1	0	100
Topiramate + PEA	1	1	0	100
Cyclobenzaprine	1	1	0	100
Neurotropin	1	0	1	0

#### 3.4.3 Continuous outcomes

Continuous outcome data were available from four studies. In the largest prospective trial of onabotulinumtoxinA (Garcia-Azorín et al., 2019), mean headache days per month decreased from 24.5 at baseline to 6.9 at weeks 20–24 (mean reduction −17.6, p < 0.001). Intense headache days decreased from 12.5 to 2.6 (−9.9, p < 0.001), and acute treatment days from 12.8 to 3.2 (−9.6, p < 0.001). In the long-term observational study (García-Iglesias et al., 2023), median headache days per month fell from 20 (IQR 10–30) at diagnosis to 3 (IQR 1–12) after a median of 80.5 months of follow-up, a median reduction of 17 days. In a single case, the ketogenic diet reduced exacerbation pain from NRS 9 to 2 at 3 months and background pain from NRS 6 to 2 at 6 months (Tereshko, 2023). Gabapentin in a paediatric case reduced VAS from 7 to 2 and HIT-6 from 54 to 13 (Panda, 2021). Temperature differences between symptomatic and contralateral scalp areas were documented in three cases (Liu, 2018), though not as a treatment outcome measure (Supplementary Table S5).

#### 3.4.4 Treatment discontinuation due to adverse events

Treatment discontinuation due to adverse events was reported for several oral preventive drugs, predominantly from the NUMITOR study (García-Iglesias et al., 2023). Gabapentin was discontinued in 21.1% (15/71) of patients due to lack of tolerability, with one additional patient in a case report stopping gabapentin because of significant side effects (Thomas, 2020). Discontinuation rates for other oral preventives were 12.9% (4/31) for amitriptyline, 13.0% (3/23) for lamotrigine, 22.2% (2/9) for pregabalin, and 25% (1/4) for carbamazepine. In contrast, no patients discontinued onabotulinumtoxinA due to adverse events across any of the included studies. Where specified, adverse effects included dizziness (gabapentin), sedation, and anticholinergic effects, though detailed event reporting was limited (Supplementary Table S6).

#### 3.4.5 Narrative synthesis of other treatments

A range of additional treatments were reported in single cases or small series, with variable efficacy. Symptomatic therapies included almotriptan (Barón et al., 2015) and ibuprofen (Liu et al., 2018), which provided significant relief. Triptans (zolmitriptan, sumatriptan) were effective in two patients with underlying superficial artery aneurysms (López-Ruiz et al., 2014). Among preventive therapies, tricyclic antidepressants (amitriptyline, nortriptyline, clomipramine) showed benefit in some cases, while mianserin, duloxetine, and mirtazapine were ineffective. Beta-blockers demonstrated only modest efficacy, with adequate response in 2/8 patients (25%) and no optimal responses (García-Iglesias et al., 2023). Anesthetic blockades (other than lidocaine) achieved adequate or optimal responses in 6/9 patients (66.7%) in the NUMITOR study. Topiramate monotherapy was adequate in 1/6 patients (16.7%), and carbamazepine monotherapy achieved optimal response in 2/4 (50%). Flunarizine showed no adequate or optimal responses (0/7). Adjunctive therapies, including PENS (Hall et al., 2021) and cryotherapy (Hall et al., 2021), provided partial or near-total relief in individual cases. Acupuncture was associated with a minor benefit in two patients (Liu et al., 2018). (Supplementary Table S7).

## 4 Discussion

This systematic review provides a descriptive synthesis of treatment outcomes in primary nummular headache (NH), including 37 studies and 1,012 patients. The available evidence suggests that onabotulinumtoxinA and gabapentin are the most frequently studied interventions. OnabotulinumtoxinA showed the most favorable reported efficacy–tolerability profile, whereas gabapentin was associated with benefit in a substantial proportion of patients but had higher discontinuation due to adverse events. However, these findings should be interpreted cautiously because the evidence base consists almost entirely of uncontrolled observational studies, case series, and case reports, with heterogeneous outcome definitions and no randomized comparative trials (Cuadrado et al., 2018; Trigo et al., 2019).

### 4.1 Synthesis of treatment outcomes

#### 4.1.1 OnabotulinumtoxinA: the most robust evidence

Among all evaluated interventions, onabotulinumtoxinA emerged as the treatment with the most consistent efficacy profile and relatively highest-quality evidence among available treatment studies base. In the NUMITOR cohort (García-Iglesias et al., 2023), onabotulinumtoxinA achieved a complete remission rate of 47.5% (19/40 patients), defined as >75% reduction in headache days, and a ≥50% responder rate of 62.5% (García-Iglesias et al., 2023). The prospective open-label PRE-NUMABOT study by García-Azorín et al., 2019 reported even higher responder rates (77.4%, 41/53 patients) and demonstrated statistically significant reductions in headache days per month (mean reduction −17.6, p < 0.001), intense headache days (−9.9, p < 0.001), and acute treatment days (−9.6, p < 0.001) (García-Azorín et al., 2019). Notably, no patients discontinued onabotulinumtoxinA due to adverse events across any included study, supporting its favorable tolerability profile. The consistency of these findings across independent cohorts, the Spanish NUMITOR registry and the Valladolid prospective trial, strengthens confidence in the efficacy of onabotulinumtoxinA for NH.

The proposed mechanism of onabotulinumtoxinA in NH aligns with the prevailing understanding of NH pathophysiology as a peripheral disorder involving epicranial structures (Trigo et al., 2019; Guerrero et al., 2012). OnabotulinumtoxinA inhibits peripheral neurotransmitter release, particularly substance P and calcitonin gene-related peptide (CGRP), thereby reducing sensitization of nociceptive fibers and interrupting the cycle of peripheral and central sensitization (García-Iglesias et al., 2022). This mechanistic rationale is supported by the observation that onabotulinumtoxinA also effectively treats other peripheral pain conditions, such as chronic migraine, where it is thought to act primarily on extracranial nociceptors. The consistent responses observed across small case series (Mathew et al., 2008; Irimia et al., 2013) and larger cohorts suggest that onabotulinumtoxinA may be particularly beneficial for patients with refractory NH or those who cannot tolerate oral preventive medications.

#### 4.1.2 Gabapentin: efficacy with tolerability limitations

Gabapentin was the most frequently evaluated preventive intervention, appearing in 12 studies with variable dosing regimens (100–1,800 mg/day). In the largest cohorts, complete remission rates ranged from 35.2% (25/71) in the NUMITOR study to 45.5% (15/33) after 5 years of follow-up in the Clar-de-Alba series (García-Iglesias et al., 2022; Clar-de-Alba et al., 2020). The ≥50% responder rate for gabapentin was 43.7% in the NUMITOR study, although Trigo and colleagues (2019) reported a higher overall response rate of 67.7% among patients receiving any oral preventive (including gabapentin) (Trigo et al., 2019). However, the most concerning finding for gabapentin was its discontinuation rate due to adverse events: 21.1% (15/71) in the NUMITOR cohort, with additional cases reported in individual case reports (García-Iglesias et al., 2022). This tolerability burden, primarily attributed to dizziness and sedation, represents a significant limitation to gabapentin’s utility in clinical practice, even when efficacy is demonstrable.

The discrepancy in reported response rates for gabapentin across studies likely reflects several factors: differences in dosage (with higher doses potentially more effective but less tolerable), variable follow-up durations, and the lack of standardized outcome definitions. In the Guerrero (2012) series, which included 72 patients, gabapentin was dosed at 800–1,800 mg/day, and 34 of 42 patients (81%) receiving any preventive treatment achieved at least 50% relief, though data for gabapentin specifically were not reported separately (Guerrero et al., 2012). The long-term outcomes reported by Clar-de-Alba (2020) suggest that among patients able to tolerate gabapentin, sustained remission is achievable, but attrition due to adverse events limits the generalizability of these findings.

#### 4.1.3 Other oral preventive medications

Amitriptyline, evaluated in seven studies (predominantly small series), showed promising efficacy in selected populations. Pellesi and colleagues (2020) reported a 66.7% (4/6) complete response rate in their Italian multicentre series, though the small sample size precludes definitive conclusions (Pellesi et al., 2020). In the NUMITOR study, amitriptyline was discontinued in 12.9% (4/31) of patients due to adverse events, primarily anticholinergic effects and sedation (García-Iglesias et al., 2022). The limited data on amitriptyline highlight a broader issue in NH literature: while tricyclic antidepressants are commonly used in clinical practice, often extrapolated from their efficacy in other chronic pain conditions, systematic evidence for their effectiveness in NH remains sparse (García-Iglesias et al., 2023).

Lamotrigine, evaluated in 23 patients in the NUMITOR study, demonstrated a more modest ≥50% responder rate of 34.8% (8/23), with a 13.0% discontinuation rate due to adverse events (García-Iglesias et al., 2022). This suggests that lamotrigine may be a second-line option for patients who cannot tolerate gabapentin or onabotulinumtoxinA, though the slow titration required to achieve therapeutic doses may limit its practicality in clinical settings.

Carbamazepine and oxcarbazepine, while associated with complete remission in individual case reports (Patil et al., 2020; Man et al., 2012), demonstrated variable efficacy in larger series. In the NUMITOR study, carbamazepine monotherapy achieved optimal response in 2 of 4 patients (50%), with one patient (25%) discontinuing due to adverse events (García-Iglesias et al., 2022). The limited sample sizes and the potential for dose-dependent adverse effects (including dizziness, ataxia, and hyponatremia) suggest that these sodium channel blockers should be reserved for cases where first-line options are ineffective or contraindicated.

Pregabalin, evaluated in the NUMITOR study (n = 9) and the Pellesi series (n = 5), showed a 50% responder rate in the pooled analysis, though discontinuation rates of 22.2% in the NUMITOR study raise similar tolerability concerns as with gabapentin (García-Iglesias et al., 2022; Pellesi et al., 2020). The Italian series reported complete disappearance of symptoms in 2 of 5 patients (40%) receiving pregabalin, suggesting that this agent may be a reasonable alternative for patients who cannot tolerate gabapentin, though comparative data are lacking.

#### 4.1.4 Other therapeutic approaches

A diverse array of other treatments was reported in single cases or small series, reflecting the uncertainty surrounding NH pathophysiology and the absence of evidence-based guidelines. Neurotropin, an immunomodulatory agent used primarily in Japan, showed promising results in three patients (two with complete remission) in the Danno series and in a single case report (Yamazaki and Kobatake, 2011; Danno et al., 2013). However, the lack of availability outside Japan and the absence of confirmatory studies limit its clinical applicability.

The response to beta-blockers was notably modest, with adequate or optimal responses in only 2 of 8 patients (25%) in the NUMITOR study, and no optimal responses were documented (García-Iglesias et al., 2022). This contrasts with the more favorable responses reported for metoprolol in two Chinese cases (Jiang et al., 2019), suggesting potential heterogeneity in treatment response based on patient characteristics or diagnostic criteria.

CGRP monoclonal antibodies (galcanezumab) have recently been reported to induce complete remission in a single case of NH occurring in a patient with migraine (López-Bravo et al., 2022). While intriguing, this finding requires replication in larger cohorts and prospective studies before recommending anti-CGRP therapies for NH without comorbid migraine. The theoretical rationale that CGRP plays a role in peripheral nociception in epicranial structures warrants further investigation (Trigo et al., 2019; García-Iglesias et al., 2023).

Interventional approaches, including anesthetic nerve blocks, demonstrated variable efficacy. In the NUMITOR study, anesthetic blockades (excluding lidocaine) achieved adequate or optimal responses in 6 of 9 patients (66.7%), suggesting that interrupting peripheral nerve transmission can be effective (García-Iglesias et al., 2022). Lidocaine injection resulted in complete pain relief in one case (Rocha-Filho, 2011), and the combination of local anesthetics with steroids was reported in other series, though systematic data are lacking. Percutaneous electrical nerve stimulation (PENS) provided near-total relief for months in a single case, and cryotherapy offered partial relief in another (Hall and Vajramani, 2021). Surgical resection of underlying superficial artery aneurysms resulted in complete and sustained remission in one patient (López-Bravo et al., 2022), underscoring the importance of excluding secondary causes in refractory cases.

#### 4.1.5 Comparison with previous literature

Previous narrative reviews on NH treatment have consistently emphasized the predominance of low-level evidence and the absence of standardized treatment guidelines (Cuadrado et al., 2018; García-Iglesias et al., 2021). Cuadrado et al. (2018) provided a comprehensive overview of NH, noting that gabapentin and onabotulinumtoxinA were the most commonly reported effective treatments, but they did not attempt a quantitative synthesis of outcomes (Cuadrado et al., 2018). Our systematic review builds upon these earlier works by providing aggregated counts of treatment response across different modalities, enabling more meaningful comparisons between therapeutic options.

Garcia-Iglesias and colleagues (2023) published the largest cohort to date (NUMITOR study, n = 183) and provided the most detailed analysis of treatment outcomes in NH, including comparative data across multiple oral preventives (García-Iglesias et al., 2022; García-Iglesias et al., 2023). Our analysis confirms and extends these findings by integrating data from smaller series and case reports, demonstrating that the efficacy estimates from the NUMITOR study are broadly consistent with aggregate data from the wider literature, though with important exceptions. For example, the high responder rates for gabapentin reported in smaller series (e.g., 100% in Rodriguez, 2015) were not replicated in the larger NUMITOR cohort, suggesting that publication bias may inflate perceived efficacy in the literature.

The long-term outcome data from Clar-de-Alba et al. (2020) and García-Iglesias et al. (2023) provide important insights into the natural history of NH and the durability of treatment responses (Clar-de-Alba et al., 2020; García-Iglesias et al., 2023). In the Garcia-Iglesias long-term series (median follow-up 80.5 months), median headache days per month decreased from 20 to 3, suggesting that the condition may improve over time regardless of treatment, though the contribution of specific interventions versus spontaneous remission remains uncertain (García-Iglesias et al., 2023). The absence of untreated control groups in all included studies represents a critical limitation of the existing evidence base, as it precludes definitive attribution of outcomes to specific treatments.

The findings of our review align with the broader literature on peripheral headache disorders, where onabotulinumtoxinA has emerged as a first-line treatment for chronic migraine and other conditions involving peripheral nociception (García-Azorín et al., 2019). The favorable safety profile of onabotulinumtoxinA, combined with its consistent efficacy across multiple cohorts, supports its consideration as a first-line or early second-line option for NH, particularly in patients who cannot tolerate oral medications or who have failed oral preventives.

#### 4.1.6 Methodological considerations and evidence gaps

The certainty of the findings is limited by the predominance of uncontrolled study designs, small sample sizes, and inconsistent outcome definitions across the included literature. Only six studies were suitable for assessment as cohort-type observational studies, and none included comparator groups, randomization, or blinded outcome assessment. Definitions of complete remission varied substantially between studies, ranging from complete pain disappearance or asymptomatic status to complete response to prophylactic therapy or “optimal response” based on a high percentage reduction in headache days. Likewise, clinically meaningful response was not uniformly defined; some studies used a standardized threshold of ≥50% improvement in headache frequency or intensity, whereas others relied on author-defined categories such as “good,” “adequate,” or “optimal” response. This variability limits direct comparison between treatments and was a major reason why quantitative synthesis was not performed.

The lack of standardized outcome measures remains a major barrier to evidence synthesis in NH. Although the International Classification of Headache Disorders provides diagnostic criteria, there is no consensus regarding the most appropriate treatment endpoints for clinical studies in NH. Future studies should adopt standardized outcomes, including monthly headache days, pain intensity using validated scales such as the Visual Analog Scale or Numeric Rating Scale, acute medication use, adverse events, treatment discontinuation, and validated measures of headache-related disability or quality of life. Consistent reporting of these outcomes would improve comparability across studies and may allow future quantitative synthesis.

The geographical distribution of included studies (predominantly Spain, followed by China, Italy, and India) raises questions about the generalizability of findings to other populations. The predominance of studies from Spain reflects the contributions of pioneering research groups in Valladolid, Alcorcón, and Madrid, who have established prospective registries and cohorts (Trigo et al., 2019; García-Iglesias et al., 2022; García-Iglesias et al., 2023; Guerrero et al., 2012; Clar-de-Alba et al., 2020). However, the lack of large-scale studies from North America, South America, Africa, and other regions suggests that NH remains underrecognized and undertreated in many settings.

The geographical concentration of the available evidence also limits generalizability. Most included studies were conducted in Spain, followed by smaller contributions from China, Italy, India, and other countries. The predominance of Spanish studies reflects the important contributions of specialized headache groups in Valladolid, Alcorcón, and Madrid, where several prospective registries and clinical cohorts have been established. However, the relative lack of large-scale studies from North America, South America, Africa, and many other regions suggests that NH may remain underrecognized and undertreated in broader clinical settings.

Potential overlap between cohorts is another important methodological concern. Several key studies were conducted in Spanish headache centers, particularly Valladolid and Alcorcón, with partially overlapping recruitment periods and recurring author groups. We assessed possible overlap using study center, author group, recruitment dates, sample size, and cohort description; however, duplicate inclusion of some patients could not be fully excluded. This is particularly relevant when interpreting descriptive counts and the apparent consistency of treatment effects across Spanish cohorts. To reduce this risk, potentially overlapping datasets were not treated as fully independent confirmatory evidence. Instead, larger or more comprehensive cohorts were prioritized for interpretation, while smaller reports were considered mainly for narrative context or unique treatment-specific information (Trigo et al., 2019; García-Iglesias et al., 2022; García-Iglesias et al., 2023; Guerrero et al., 2012; García-Azorín et al., 2019).

Clinical heterogeneity is also important. Some cases classified as primary NH may have had an unrecognized traumatic component. Although this review focused on primary NH and excluded studies dedicated solely to secondary or post-traumatic NH, trauma history was not consistently assessed or reported across all included cohorts. This may be especially relevant in older patients, in whom minor or remote head trauma may be underreported or difficult to identify retrospectively. Trauma-associated NH may differ from spontaneous primary NH in pathophysiology, clinical course, and treatment response. Therefore, incomplete assessment of trauma history may have influenced the estimated treatment responses. Future studies should systematically document previous head trauma and report outcomes separately for spontaneous primary NH and trauma-associated cases.

Another source of heterogeneity is the possible presence of migraine-like features in some patients with NH. Although NH is classified as a distinct primary headache disorder, some patients may have migrainous symptoms, comorbid migraine, or overlapping peripheral and central nociceptive mechanisms. This could partly explain reported responses to treatments commonly used for migraine, including triptans, beta-blockers, topiramate, CGRP monoclonal antibodies, and onabotulinumtoxinA. For example, galcanezumab response has been reported in a patient with coexisting migraine, and responses to triptans or migraine preventives may reflect an overlapping migraine phenotype rather than NH-specific efficacy alone. Because migraine comorbidity and migrainous features were not consistently reported, these observations should be interpreted cautiously. Future studies should document migraine history, migrainous symptoms, and phenotype-specific treatment responses.

Age may further influence treatment choice and tolerability. Because NH often affects middle-aged and older adults, adverse effects from oral preventives such as gabapentin may be particularly relevant. In this review, gabapentin was associated with discontinuation due to poor tolerability in 21.1% of patients in the NUMITOR cohort. However, the included studies did not consistently provide patient-level data linking age, gabapentin dose, adverse events, and discontinuation. As a result, a formal age-stratified analysis of gabapentin tolerability or dose-response could not be performed. This represents an important limitation of the current evidence. Clinically, gabapentin should be prescribed cautiously in older patients, with individualized dosing, slow titration, and close monitoring for dizziness, sedation, gait instability, cognitive slowing, and treatment discontinuation.

#### 4.1.7 Future research directions

The findings of this systematic review highlight several critical priorities for future research in primary NH. First, prospective comparative trials with standardized outcome measures are urgently needed to establish definitive treatment guidelines. Given the rarity of NH, such trials may require multicentre, international collaborations to achieve adequate sample sizes. The success of the Spanish NUMITOR registry and the Italian RegistRare Network demonstrates the feasibility of collaborative approaches to studying rare headache disorders.

Second, randomized controlled trials comparing onabotulinumtoxinA with gabapentin or placebo would provide the highest level of evidence for treatment efficacy. Our findings suggest that onabotulinumtoxinA has superior tolerability and at least comparable efficacy to gabapentin, but head-to-head comparisons are lacking. Such trials should incorporate standardized outcome measures, including monthly headache days, responder rates, and quality-of-life assessments, with adequate sample sizes to detect clinically meaningful differences.

Third, mechanistic studies are needed to better understand the pathophysiology of NH and identify therapeutic targets. The observation that onabotulinumtoxinA and nerve blocks are effective supports a peripheral mechanism, but the variable response to sodium channel blockers and calcium channel modulators suggests that central sensitization may also play a role. Advanced neuroimaging, quantitative sensory testing, and biomarker studies could help elucidate the relative contributions of peripheral and central mechanisms.

Fourth, the role of CGRP monoclonal antibodies in NH warrants further investigation. The single case report of galcanezumab responsiveness suggests that CGRP may be involved in NH pathophysiology, but larger studies are needed to confirm this finding and determine whether anti-CGRP therapies have a role in NH without comorbid migraine.

Fifth, long-term outcome studies with extended follow-up are needed to determine the durability of treatment responses and the natural history of NH. The available long-term data suggest that NH may improve over time, but the contributions of specific treatments versus spontaneous remission remain unclear. Prospective cohort studies with systematic follow-up and documentation of treatment changes over time would help address this question.

Finally, the development of validated patient-reported outcome measures specific to NH would facilitate future research and clinical care. While generic headache measures can be applied, an NH-specific measure would capture the unique features of this condition, including the well-circumscribed pain, sensory changes, and impact on daily activities.

## 5 Conclusion

In conclusion, this systematic review provides a structured descriptive synthesis of treatment outcomes in primary nummular headache, demonstrating that onabotulinumtoxinA has the most robust evidence for efficacy and tolerability, with high responder rates (62.5%–77.4%) and no reported discontinuations due to adverse events. Gabapentin is effective in a substantial proportion of patients (35.2%–45.5% complete remission) but is limited by a 21.1% discontinuation rate due to adverse events. Other oral preventives, including amitriptyline, lamotrigine, and carbamazepine, show variable efficacy and require further study. The overall quality of evidence is limited by the predominance of uncontrolled designs, heterogeneous outcome definitions, and small sample sizes. Standardized outcome definitions, prospective comparative studies, and international collaborations are needed to establish definitive treatment guidelines and improve outcomes for patients with this rare but disabling headache disorder.

## Data Availability

The datasets presented in this study can be found in online repositories. The names of the repository/repositories and accession number(s) can be found in the article/Supplementary Material.

## Glossary

NH: Nummular Headache
ICHD: International Classification of Headache Disorders
ICHD-2: International Classification of Headache Disorders, 2nd Edition
ICHD-3β: International Classification of Headache Disorders, 3rd Edition (Beta Version)
ICHD-3: International Classification of Headache Disorders, 3rd Edition
CGRP: Calcitonin Gene-Related Peptide
NSAIDs: Nonsteroidal Anti-Inflammatory Drugs
PRISMA: Preferred Reporting Items for Systematic Reviews and Meta-Analyses
PROSPERO: International Prospective Register of Systematic Reviews
PICO: Population, Intervention, Comparator, Outcome
JBI: Joanna Briggs Institute
NOS: Newcastle–Ottawa Scale
SD: Standard Deviation
SE: Standard Error
MC: Mean Change
MD: Mean Difference
CI: Confidence Interval
RD: Risk Difference
VAS: Visual Analog Scale
NRS: Numeric Rating Scale
IQR: Interquartile Range
Tx: Treatment
NR: Not Reported
NS: Not Specified
PRN: Pro Re Nata (as needed)
TID: Three Times Daily
OnabotA: OnabotulinumtoxinA
PEA: Palmitoylethanolamide
VLCKD: Very-Low-Calorie Ketogenic Diet
PENS: Percutaneous Electrical Nerve Stimulation
F: Female
