# Supplementary material for: Treatment outcomes in primary nummular headache: a systematic review
Source: Front Pharmacol. 2026 Aug 20;17:1870131. doi: 10.3389/fphar.2026.1870131 (PMC13540074; doi:10.3389/fphar.2026.1870131)
Supplement: Supplementary file 1 [file Supplementaryfile1.docx]

# 1. **Research strategy terms**

**Table 1 sup. research strategy details.**

| **Database** | **Date Coverage** | **Search Strategy** |
| --- | --- | --- |
| PubMed | 2002–present | ("nummular headache"[Title/Abstract] OR "coin-shaped headache"[Title/Abstract] OR "coin shaped headache"[Title/Abstract] OR "coin-shaped cephalalgia"[Title/Abstract] OR "coin shaped cephalalgia"[Title/Abstract]) AND (therap*[Title/Abstract] OR treatment*[Title/Abstract] OR management[Title/Abstract] OR outcome*[Title/Abstract] OR response[Title/Abstract] OR remission[Title/Abstract] OR gabapentin[Title/Abstract] OR pregabalin[Title/Abstract] OR "botulinum toxin"[Title/Abstract] OR onabotulinumtoxinA[Title/Abstract] OR NSAID*[Title/Abstract] OR indomethacin[Title/Abstract] OR amitriptyline[Title/Abstract] OR carbamazepine[Title/Abstract] OR "nerve block"[Title/Abstract]) |
| Embase (Ovid) |  | (exp nummular headache/ OR (nummular headache OR coin-shaped headache OR coin shaped headache OR coin-shaped cephalalgia OR coin-shaped cephalalgia).ti, ab.) AND (exp treatment outcome/ OR exp drug therapy/ OR exp pharmacotherapy/ OR exp therapy/ OR (treat* OR therap* OR manag* OR outcome* OR response OR remission OR prophyla* OR prevent*).ti, ab. OR exp gabapentin/ OR exp pregabalin/ OR exp botulinum toxin A/ OR exp onabotulinumtoxinA/ OR exp nonsteroid antiinflammatory agent/ OR exp indomethacin/ OR exp amitriptyline/ OR exp tricyclic antidepressant/ OR exp carbamazepine/ OR exp oxcarbazepine/ OR exp lamotrigine/ OR exp topiramate/ OR exp nerve block/ OR exp local anesthetic agent/ OR exp surgery/ OR (gabapentin OR pregabalin OR botulinum toxin OR onabotulinumtoxinA OR BoNT-A OR NSAID* OR non-steroidal anti-inflammatory OR indomethacin OR amitriptyline OR tricyclic antidepressant* OR carbamazepine OR oxcarbazepine OR lamotrigine OR topiramate OR nerve block OR local anesthetic OR surgical OR resection).ti, ab.) AND 2002:current |
| Scopus |  | (TITLE-ABS-KEY("nummular headache" OR "coin-shaped headache" OR "coin shaped headache" OR "coin-shaped cephalalgia" OR "coin shaped cephalalgia")) AND (TITLE-ABS-KEY(treat* OR therap* OR manag* OR outcome* OR response OR remission OR prophyla* OR prevent* OR gabapentin OR pregabalin OR "botulinum toxin" OR onabotulinumtoxinA OR "BoNT-A" OR nsaid* OR "non-steroidal anti-inflammatory" OR indomethacin OR amitriptyline OR "tricyclic antidepressant*" OR carbamazepine OR oxcarbazepine OR lamotrigine OR topiramate OR "nerve block" OR "local anesthetic" OR surgical OR resection)) AND PUBYEAR > 2001 |
| Web of Science |  | TS=("nummular headache" OR "coin-shaped headache" OR "coin shaped headache" OR "coin-shaped cephalalgia" OR "coin shaped cephalalgia") AND TS=(treat* OR therap* OR manag* OR outcome* OR response OR remission OR prophyla* OR prevent* OR gabapentin OR pregabalin OR "botulinum toxin" OR onabotulinumtoxinA OR "BoNT-A" OR nsaid* OR "non-steroidal anti-inflammatory" OR indomethacin OR amitriptyline OR "tricyclic antidepressant*" OR carbamazepine OR oxcarbazepine OR lamotrigine OR topiramate OR "nerve block" OR "local anesthetic" OR surgical OR resection) AND PY=(2002–2026) |
| Cochrane Library |  | ("nummular headache" OR "coin-shaped headache" OR "coin shaped headache" OR "coin-shaped cephalalgia" OR "coin shaped cephalalgia"): ti ,ab,kw AND (treat* OR therap* OR manag* OR outcome* OR response OR remission OR prophyla* OR prevent* OR gabapentin OR pregabalin OR "botulinum toxin" OR onabotulinumtoxinA OR "BoNT-A" OR nsaid* OR "non-steroidal anti-inflammatory" OR indomethacin OR amitriptyline OR "tricyclic antidepressant*" OR carbamazepine OR oxcarbazepine OR lamotrigine OR topiramate OR "nerve block" OR "local anesthetic" OR surgical OR resectio n ):ti,ab,kw |

**Abbreviations:** ti, title; ab, abstract; kw, keywords; TS, topic; exp, exploded subject heading; MeSH, Medical Subject Headings; Emtree, Embase subject headings; NSAID, non-steroidal anti-inflammatory drug; BoNT-A, botulinum toxin type A.

**Notes:** Search strategies were adapted to the indexing system of each database (e.g., MeSH in PubMed and Emtree in Embase). Free-text terms and controlled vocabulary were combined where applicable. Truncation symbols (*) were used to capture word variations. The search was limited to studies published from 2002 onwards, corresponding to the first description of nummular headache.

# 2. **Results**

**Table 2 sup.Quality Assessment details:**

| **Study ID** | **Design** | **Tool** | **Score** | **Comments** |
| --- | --- | --- | --- | --- |
| Barón, 2015 | Case report | JBI for Case Reports | 7/8 | Patient demographics, clinical history, treatment, and outcomes were clearly described; no adverse events were reported; follow-up was not specified. |
| Camacho‑Velasquez, 2016 | Case report | JBI for Case Reports | 8/8 | Detailed history, examination, imaging, biopsy, treatment, and response; clear description. |
| Chen, 2013 | Case series (n=5) | JBI for Case Series | 7/10 | Clear inclusion criteria, consecutive cases from a larger cohort, and outcomes reported; lacks statistical analysis and long-term follow-up. |
| Chirchiglia, 2020 | Case report | JBI for Case Reports | 7/8 | Good description of case, treatment, and 3‑year follow-up; missing some details on exacerbation frequency. |
| Clar-de-Alba, 2020 | Retrospective case series (long-term) | NOS (cohort) | 5/9 | Selection: ★★ (representative, but no non‑exposed). Outcome: ★★★ (long follow-up, minimal loss). |
| Danno, 2013 | Case series (n=3) | JBI for Case Reports (adapted) | 6/8 | Cases well described; response to Neurotropin noted; follow-up limited in one case. |
| Evans, 2005 | Case report | JBI for Case Reports | 7/8 | Detailed clinical history, expert commentary, follow-up with MRI, and treatment. |
| Garcia-Azorín, 2019 | Prospective, open-label, single-arm | NOS (cohort) | 6/9 | Selection: ★★★ (representative exposed, exposure secure). Comparability: ★ (no comparison group). Outcome: ★★★ (record linkage, follow-up adequate). |
| García-Iglesias, 2023 (JCM) | Ambispective cohort (NUMITOR) | NOS (cohort) | 6/9 | Selection: ★★★ (clearly defined, secure exposure). Outcome: ★★★ (standardised diaries, adequate follow-up). |
| García-Iglesiasas, 2023_ (Cephalalgia) | Observational, ambispective | NOS (cohort) | 6/9 | Selection: ★★★ (consecutive, representative). Outcome: ★★★ (record linkage, long follow-up, attrition accounted). |
| Guerrero, 2011 | Case series (n=6) | JBI for Case Series | 7/10 | Bifocal NH, descriptive, treatment responses noted, no systematic follow-up. |
| Guerrero, 2012 | Case series (n=72) | JBI for Case Series | 9/10 | Well-defined demographic and clinical features, consecutive patients, clear reporting of outcomes; follow-up limited for treatment response. |
| Hall, 2021 | Case report | JBI for Case Reports | 8/8 | Comprehensive description, cumulative treatment effect, follow-up over 4 years, and clear outcomes. |
| Irimia, 2013 | Case report | JBI for Case Reports | 8/8 | Detailed case, histopathology, treatment with onabotA, prolonged follow-up, clear outcomes. |
| Iwanowski, 2013 | Case report | JBI for Case Reports | 6/8 | Basic description: treatment response unclear; follow-up limited. |
| Jiang, 2019 | Case series (n=2) | JBI for Case Reports (adapted) | 7/8 | Detailed case descriptions, follow-up 6‑12 months, cand lear treatment response. |
| Liu, 2018 | Case series (n=3) | JBI for Case Reports (adapted) | 6/8 | Cases described, temperature changes reported, but treatment response not systematic. |
| López‑Bravo, 2022 | Case report | JBI for Case Reports | 8/8 | Clear description, treatment with galcanezumab, detailed timeline, and response. |
| Lopez‑Mesonero, 2014 | Case report | JBI for Case Reports | 7/8 | Good description, imaging, no treatment evaluated; follow-up not specified. |
| López‑Ruiz, 2014 | Case series (n=2) | JBI for Case Reports (adapted) | 8/8 | Detailed cases, imaging, treatment response (surgery, triptans), follow-up. |
| Man, 2012 | Case report | JBI for Case Reports | 7/8 | Clear description, neurological findings, treatment with carbamazepine, follow-up 6 months. |
| Mathew, 2008 | Case series (n=4) | JBI for Case Series | 8/10 | Consecutive? Not explicitly stated, but a clear baseline and outcomes, no statistical analysis. |
| Mulero, 2013 | Case series (n=3) | JBI for Case Reports (adapted) | 6/8 | Cases described, treatment responses noted, follow-up not detailed. |
| Panda, 2021 | Case report | JBI for Case Reports | 7/8 | Pediatric case, clear description, treatment response with gabapentin, follow-up 3 months. |
| Pareja, 2004 | Case series (n=14) | JBI for Case Series | 8/10 | Clear inclusion criteria, consecutive patients, complete outcomes, but no statistical analysis; follow-up not uniformly long. |
| Patel, 2020 | Case report | JBI for Case Reports | 7/8 | Clear description, treatment with oxcarbazepine, rechallenge response, follow-up 1 year. |
| Pellesi, 2020 | Retro‑prospective multi‑centre | NOS (cohort) | 5/9 | Selection: ★★★ (representative, exposure from records). Outcome: ★★ (outcome by record, follow-up not systematically described). |
| Porta‑Etessam, 2010 | Case report | JBI for Case Reports | 7/8 | Multifocal NH with trophic changes; follow-up 4 months; no treatment evaluated. |
| Rammohan, 2016 | Case series (n=29) | JBI for Case Series | 7/10 | Consecutive patients, descriptive only, no treatment evaluated; follow-up not for intervention. |
| Robbins, 2010 | Case report | JBI for Case Reports | 7/8 | Good description, menstrual association, treatment with cyclobenzaprine, follow-up not specified. |
| Rocha‑Filho, 2011 | Case report | JBI for Case Reports | 7/8 | Clear case, bifocal, treatment with lidocaine, response, follow-up not detailed. |
| Rodriguez, 2015 | Case report | JBI for Case Reports | 8/8 | Multifocal NH, pressure pain sensitivity map, gabapentin response, follow-up 20 months. |
| Ruscheweyh, 2010 | Case series (n=6) | JBI for Case Series | 7/10 | Cases well described, variable treatments, follow-up not systematic; lacks statistical analysis. |
| Tereshko, 2023 | Case report | JBI for Case Reports | 8/8 | Detailed case, sequential treatments (diet, onabotA), clear outcomes, and follow-up. |
| Thomas, 2020 | Case report | JBI for Case Reports | 8/8 | Comprehensive case, diagnostic work-up, treatment, side effects, follow-up, adjunctive therapies. |
| Trigo, 2019 | Observational, descriptive | NOS (cohort) | 5/9 | Selection: ★★★ (consecutive, representative). Outcome: ★★ (outcome by record, follow-up not detailed). |
| Yamazaki, 2011 | Case report | JBI for Case Reports | 7/8 | Clear case, treatment with Neurotropin, follow-up 12 weeks, good response. |

¹ Quality assessment tools:

- JBI for Case Reports (Joanna Briggs Institute Critical Appraisal Checklist for Case Reports): 8 items assessing patient demographics, clinical history, examination findings, diagnostic tests, interventions, outcomes, adverse events, and follow‑up. Maximum score = 8.
- JBI for Case Series (JBI Critical Appraisal Checklist for Case Series): 10 items evaluating inclusion criteria, measurement of condition, consecutive inclusion, complete case description, outcome reporting, and statistical analysis. Maximum score = 10.
- NOS for Cohort Studies (Newcastle–Ottawa Scale): 8 items across three domains – Selection (maximum 4 stars), Comparability (maximum 2 stars), Outcome (maximum 3 stars). Total maximum = 9 stars.

² Scoring interpretation:

- For JBI checklists, higher scores indicate more complete reporting. Scores are presented as total items met (e.g., 7/8).
- For NOS, star ratings are shown in the Comments column (e.g., Selection: ★★★) and the numeric score represents the total stars awarded.

³ Adapted tools: Studies labelled “JBI for Case Reports (adapted)” applied the JBI case report checklist to each individual within a small case series, consistent with the original tool’s design for single cases.

⁴ Scores reflect the original published quality assessments. No changes were made to the scores presented in this table; the footnotes serve only to clarify the methodology of the assessment.

**Table 3 sup: Complete Remission (Primary Outcome) – All Treatments**

| **Treatment** | **Study (Year)** | **N treated** | **N complete remission** | **Proportion (%)** | **Definition of complete remission** |
| --- | --- | --- | --- | --- | --- |
| Gabapentin | Clar‑de‑Alba, 2020 | 33 | 15 | 45.5 | “Asymptomatic with complete response to prophylactic treatment” after 5 years of follow-up |
| Gabapentin | García‑Iglesias, 2023 (NUMITOR) | 71 | 25 | 35.2 | Optimal response (>75% reduction in headache days per month) |
| Gabapentin | Rodriguez, 2015 | 1 | 1 | 100 | Complete pain remission |
| Gabapentin | Chen, 2013 (Case 2,5) | 2 | 2 | 100 | Rapid abolishment, no recurrence 1 year |
| Gabapentin | Mulero, 2013 (Patient 1) | 1 | 1 | 100 | Complete pain remission |
| OnabotulinumtoxinA | García‑Iglesias, 2023 (NUMITOR) | 40 | 19 | 47.5 | Optimal response (>75% reduction) |
| OnabotulinumtoxinA | Tereshko, 2023 | 1 | 1 | 100 | Exacerbation pain abolished, background disappeared |
| Neurotropin | Danno, 2013 (Case 1 & 3) | 3 | 2 | 66.7 | Pain disappearance (Case 1: 1 month; Case 3: 5 days) |
| Neurotropin | Yamazaki, 2011 | 1 | 1 | 100 | No recurrence after 2 months |
| Metoprolol | Jiang, 2019 (Case 1) | 1 | 1 | 100 | Complete freedom from headache |
| Galcanezumab | López‑Bravo, 2022 | 1 | 1 | 100 | Complete remission within 2 weeks |
| Oxcarbazepine | Patel, 2020 | 1 | 1 | 100 | Complete improvement |
| Carbamazepine | Man, 2012 | 1 | 1 | 100 | Pain resolved, pain‑free at 6 months |
| Indomethacin | Mulero, 2013 (Patient 2) | 1 | 1 | 100 | Total relief of pain |
| Lidocaine injection | Rocha‑Filho, 2011 | 1 | 1 | 100 | Complete pain relief |
| Surgery (aneurysm resection) | López‑Ruiz, 2014 (Patient 1) | 1 | 1 | 100 | Asymptomatic after 8 months |

**Table 4 sup: Clinically Meaningful Response (≥50% Improvement) – All Treatments**

| **Treatment** | **Study (Year)** | **N treated** | **N with ≥50% improvement** | **Proportion (%)** | **Response definition** |
| --- | --- | --- | --- | --- | --- |
| Gabapentin | Garcia‑Iglesias, 2023 (NUMITOR) | 71 | 31 (6+25) | 43.7 | ≥50% reduction in headache days |
| Gabapentin | Guerrero, 2012 | 37 | not reported separately | – | At least 50% relief in 34/42 on preventatives overall |
| Gabapentin | Trigo, 2019 | 107 | 86 (overall) | 67.7 | >50% reduction in headache days (overall) |
| OnabotulinumtoxinA | Garcia‑Iglesias, 2023 (NUMITOR) | 40 | 25 | 62.5 | ≥50% responder rate |
| OnabotulinumtoxinA | Garcia‑Azorín, 2019 | 53 | 41 | 77.4 | ≥50% responder rate (weeks 20‑24) |
| OnabotulinumtoxinA | Tereshko, 2023 | 1 | 1 | 100 | Exacerbation abolished |
| OnabotulinumtoxinA | Irimia, 2013 | 1 | 1 | 100% | “Dramatic reduction in headache frequency, with only 1-2 headache days/month” |
| OnabotulinumtoxinA | Mathew, 2008 | 4 | 4 | 100% | “Reduction in pain, allodynia, and/or paresthesia” |
| Pregabalin | Pellesi, 2020 | 5 | 2 | 40 | Complete disappearance (stricter endpoint) |
| Amitriptyline | Pellesi, 2020 | 6 | 4 | 66.7 | Complete disappearance |
| Lamotrigine | Garcia‑Iglesias, 2023 (NUMITOR) | 23 | 8 | 34.8 | ≥50% response (3 adequate + 5 optimal) |
| Carbamazepine | Guerrero, 2011 (Patient 3) | 1 | 1 | 100 | “Until achieving remission” |
| Carbamazepine | Panda, 2021 | 1 | partial only | – | Partial, then add‑on gabapentin |
| Topiramate | Ruscheweyh, 2010 (Patient 5) | 1 | 1 | 100 | Reduced intensity 3‑5, frequency 3‑5/day |
| Amitriptyline | Clar‑de‑Alba, 2020 | 21 | not reported separately | – | Partial responses in 23, complete in 10 overall |
| Anesthetic blocks | Rocha‑Filho, 2011 | 1 | 1 | 100 | Lidocaine complete relief |
| Ketogenic diet (VLCKD) | Tereshko, 2023 | 1 | 1 | 100 | Exacerbation NRS 9→2, background 6→2 |
| PENS | Hall, 2021 | 1 | 1 | 100 | Near‑total pain relief for months |
| Metoprolol | Jiang, 2019 (Case 2) | 1 | 1 | 100 | Mild pain (2‑3/10) |
| Neurotropin | Danno, 2013 (Case 2) | 1 | 0 | 0 | Slight relief only |
| Topiramate | Barón, 2015 | 1 | 1 | 100 | The study reports “significant reduction in pain attacks (two per month) and their intensity (5 of 10)”. Complete remission is not claimed. |
| Pregabalin | Camacho‑Velasquez, 2016 | 1 | 1 | 100 | “Good response to the pain” – no mention of complete resolution. |
| Topiramate + PEA | Chirchiglia, 2020 | 1 | 1 | 100 | “Occasional mild pain” – not complete remission. |
| Cyclobenzaprine | Robbins, 2010 | 1 | 1 | 100 | “Intensity reduced by at least 80%” – not complete remission." |

**Table 5 sup: Continuous Outcomes (Mean Change in Headache Days / Pain Intensity)**

| **Treatment** | **Study** | **Outcome** | **Baseline Mean (SD)** | **Post-Treatment Mean (SD)** | **Mean Change (SD)** | **p-value** | **Time Point** |
| --- | --- | --- | --- | --- | --- | --- | --- |
| OnabotulinumtoxinA | Garcia‑Azorín, 2019 | Headache days/month | 24.5 | 6.9 (weeks 20‑24) | −17.6 | <0.001 | Weeks 20-24 |
| OnabotulinumtoxinA | Garcia‑Azorín, 2019 | Intense headache days/month | 12.5 | 2.6 | −9.9 | <0.001 | Weeks 20-24 |
| OnabotulinumtoxinA | Garcia‑Azorín, 2019 | Acute treatment days/month | 12.8 | 3.2 | −9.6 | <0.001 | Weeks 20-24 |
| Various preventives | Garcia‑Iglesias, 2023 (long‑term) | Headache days/month | 20 (IQR 10‑30) | 3 (IQR 1‑12) | −17 (median) | NR | Median 80.5 months follow-up |
| Ketogenic diet | Tereshko, 2023 | Exacerbation NRS | 9 | 2 (3 months) | −7 | NR | NR |
| Ketogenic diet | Tereshko, 2023 | Background pain NRS | 6 | 2 (6 months) | −4 | NR | NR |
| Gabapentin | Panda, 2021 | VAS | 7 | 2 | −5 | NR | NR |
| Gabapentin | Panda, 2021 | HIT‑6 | 54 | 13 | −41 | NR | NR |
| Gabapentin | Liu, 2018 (Case 1) | Scalp temperature (symptomatic area) | 35.5-36.1°C | Not reported | N/A | N/A | N/A (temperature difference noted) |
| Acupuncture/gabapentin | Liu, 2018 (Case 2) | Scalp temperature (symptomatic area) | 37.3-37.4°C | Not reported | N/A | N/A | N/A (temperature difference noted) |
| Ibuprofen/acupuncture | Liu, 2018 (Case 3) | Scalp temperature (symptomatic area) | 37.3-37.5°C | Not reported | N/A | N/A | N/A (temperature difference noted) |

**Table 6 sup: Treatment Discontinuation Due to Adverse Events**

| **Treatment** | **Study** | **N treated** | **Discontinued due to AE** | **Proportion (%)** | **Main AEs** |
| --- | --- | --- | --- | --- | --- |
| Gabapentin | Garcia‑Iglesias, 2023 (NUMITOR) | 71 | 15 | 21.1 | Not specified (lack of tolerability) |
| Gabapentin | Thomas, 2020 | 1 | 1 | 100 | Significant side effects (voluntary stop) |
| Gabapentin | López‑Bravo, 2022 | 1 | 0 | 0 | Dizziness at 900 mg but continued |
| Amitriptyline | García‑Iglesias, 2023 (NUMITOR) | 31 | 4 | 12.9 | Not specified |
| Lamotrigine | Garcia‑Iglesias, 2023 (NUMITOR) | 23 | 3 | 13 | Not specified |
| Pregabalin | Garcia‑Iglesias, 2023 (NUMITOR) | 9 | 2 | 22.2 | Not specified |
| Carbamazepine | Garcia‑Iglesias, 2023 (NUMITOR) | 4 | 1 | 25 | Not specified |

**Table 7 sup: Narrative Synthesis of Other Treatments (Not Included in Previous Tables)**

| **Treatment** | **Study (Year)** | **N treated** | **Key Findings** |
| --- | --- | --- | --- |
| Almotriptan (symptomatic) | Barón, 2015 | 1 | Significant relief of pain attacks |
| Mianserin | Iwanowski, 2013 | 1 | No effect |
| Bupivacaine injections | Iwanowski, 2013 | 1 | No effect on NH (relieved occipital neuralgia) |
| Duloxetine | Ruscheweyh, 2010 | 1 | No clear response |
| Amitriptyline | Ruscheweyh, 2010 | 1 | No response |
| Clomipramine | Cited in Ruscheweyh, 2010 | 1 | Pain relief in one case (literature) |
| Nortriptyline | Cited in Ruscheweyh, 2010 | 1 | Pain reduction in one case |
| Valproate | Mentioned in review (no new data) | – | Not used in included studies |
| Flunarizine | Garcia‑Iglesias, 2023 (NUMITOR) | 7 | No patient achieved adequate or optimal response |
| Beta‑blockers (propranolol, etc.) | Garcia‑Iglesias, 2023 (NUMITOR) | 8 | 2 (25%) adequate response, 0 optimal |
| Anesthetic blockades (other than lidocaine) | Garcia‑Iglesias, 2023 (NUMITOR) | 9 | 1 (11%) adequate, 5 (56%) optimal |
| Mirtazapine | Garcia‑Iglesias, 2023 (NUMITOR) | 1 | No response |
| Topiramate (monotherapy) | Garcia‑Iglesias, 2023 (NUMITOR) | 6 | 1 (16.7%) adequate response |
| Carbamazepine (as monotherapy) | Garcia‑Iglesias, 2023 (NUMITOR) | 4 | 2 (50%) optimal response |
| Pregabalin (as monotherapy) | Garcia‑Iglesias, 2023 (NUMITOR) | 9 | 1 (11%) optimal, 1 adequate |
| Triptans (zolmitriptan, sumatriptan) | López‑Ruiz, 2014 | 2 | Both patients reported a response (transient) |
| Local anesthetic + steroid injections | Hall, 2021 | 1 | Unsuccessful |
| Cryotherapy | Hall, 2021 | 1 | Partial relief for 3 months |
| Acupuncture | Liu, 2018 (Cases 2 & 3) | 2 | Minor benefit |
| Ibuprofen (symptomatic) | Liu, 2018 (Case 3) | 1 | Good response |
| Venlafaxine | Tereshko, 2023 | 1 | Slight benefit on intensity only |
| Paracetamol | Tereshko, 2023 | 1 | Partial response |
| NSAIDs (unspecified) | Multiple studies | Various | Variable response, often partial |

**Figure 1 sup: Complete remission histogram.**

**Figure 2 sup: ≥50% response histogram**
